# Molecular Signature of High Yield (Growth) Influenza A Virus Reassortants Prepared as Candidate Vaccine Seeds

**DOI:** 10.1371/journal.pone.0065955

**Published:** 2013-06-11

**Authors:** Manojkumar Ramanunninair, Jianhua Le, Shiroh Onodera, Andrew A. Fulvini, Barbara A. Pokorny, Jeanmarie Silverman, Rene Devis, Jennifer M. Arroyo, Yu He, Alex Boyne, Jayati Bera, Rebecca Halpin, Erin Hine, David J. Spiro, Doris Bucher

**Affiliations:** 1 Department of Microbiology and Immunology, New York Medical College, Valhalla, New York, United States of America; 2 Department of Infectious Disease, J. Craig Venter Institute, Rockville, Maryland, United States of America; 3 Influenza, SARS and Related Viral Respiratory Diseases Branch, Division of Microbiology and Infectious Diseases, NIAID/NIH/DHHS, Bethesda, Maryland, United States of America; Centers for Disease Control and Prevention, United States of America

## Abstract

**Background:**

Human influenza virus isolates generally grow poorly in embryonated chicken eggs. Hence, gene reassortment of influenza A wild type (wt) viruses is performed with a highly egg adapted donor virus, A/Puerto Rico/8/1934 (PR8), to provide the high yield reassortant (HYR) viral ‘seeds’ for vaccine production. HYR must contain the hemagglutinin (HA) and neuraminidase (NA) genes of wt virus and one to six ‘internal’ genes from PR8. Most studies of influenza wt and HYRs have focused on the HA gene. The main objective of this study is the identification of the molecular signature in all eight gene segments of influenza A HYR candidate vaccine seeds associated with high growth *in ovo.*

**Methodology:**

The genomes of 14 wt parental viruses, 23 HYRs (5 H1N1; 2, 1976 H1N1-SOIV; 2, 2009 H1N1pdm; 2 H2N2 and 12 H3N2) and PR8 were sequenced using the high-throughput sequencing pipeline with big dye terminator chemistry.

**Results:**

Silent and coding mutations were found in all internal genes derived from PR8 with the exception of the M gene. The M gene derived from PR8 was invariant in all 23 HYRs underlining the critical role of PR8 M in high yield phenotype. None of the wt virus derived internal genes had any silent change(s) except the PB1 gene in X-157. The highest number of recurrent silent and coding mutations was found in NS. With respect to the surface antigens, the majority of HYRs had coding mutations in HA; only 2 HYRs had coding mutations in NA.

**Significance:**

In the era of application of reverse genetics to alter influenza A virus genomes, the mutations identified in the HYR gene segments associated with high growth *in ovo* may be of great practical benefit to modify PR8 and/or wt virus gene sequences for improved growth of vaccine ‘seed’ viruses.

## Introduction

Influenza A viruses (IAV) are single stranded, negative sense RNA viruses belonging to the family *Orthomyxoviridae* [1]. IAV have genomes comprised of eight segments which encode thirteen proteins [2]–[6]. IAV causes both seasonal epidemics and infrequently pandemics as occurred in 1918, 1957, 1968, 1977 and 2009. IAV undergo antigenic drift in the HA and to a lesser extent in the NA genes as a consequence of the high mutation rates introduced into the genome by the viral polymerase complex during replication [7], [8]. As a result, frequent updates of the influenza vaccine composition are necessary to ensure that the antigenic properties are as closely matched as possible to the most prevalent antigenic variants circulating in the population [9], [10].

Vaccination is the primary method of influenza prevention and control [11], [12]. The first inactivated influenza virus vaccine was produced in the 1940s using wild type (wt) virus [13]. Since wt viruses generally grow to low titer in embryonated chicken eggs and efficient vaccine production depends on a virus ‘seed’ with the ability to grow to high titer, high yield reassortants (HYRs) were developed for type A influenza vaccine production [14]. To date the majority of IAV used for preparation of inactivated vaccines have been HYRs [15], [16].

Currently there are two techniques to generate HYRs or HGRs (High Growth Reassortants); the ‘classical’ reassortment method and the reverse genetics approach. In the classical method, HYRs are generated by co-inoculation of two viruses *in ovo*, namely a wt virus and a hy donor virus (A/Puerto Rico/8/1934 (PR8) or an H3N2 HYR with six PR8 genes encoding the ‘internal’ proteins). HYRs must contain genes for the two surface glycoproteins, HA and NA, of the wt virus and one or more of the ‘internal’ genes from PR8 or its derivatives. The classical reassortment method is based on the selection of HYRs from a potential pool of 254 reassortants (256 total progeny minus 2 parents) via negative selection using antibodies and/or antisera against the hy donor HA and NA; thus reducing the pool to 64 candidates [17], [18]. Positive selection occurs naturally in the egg host with the highest growing reassortant best adapted to growth *in ovo,* out-replicating lower growing reassortants. HYR candidates are then cloned by limiting dilution [14], [15], [18]. In the reverse genetics approach, a reassortant virus with a predefined gene constellation (donor:wt) is generated in mammalian cells using a plasmid system with no negative selection process involved [19]–[23]. The reverse genetics reassortants are generated to have a fixed gene constellation, generally 6∶2 [six internal genes derived from PR8 and the genes for the two surface glycoproteins, HA (either native or modified) and NA from the wt virus] [24].

Most genetic characterization studies of influenza wt and reassortant viruses have focused on the importance of HA coding changes [25]–[29]. Previous studies involving one or two HYRs have shown that HYRs deriving one or more of the PR8 internal genes grow better in eggs than the original low yielding wt virus [30]–[37]. However, large scale sequence analysis of IAV HYRs belonging to different subtypes has not been performed to study the egg adaptive changes occurring in the internal genes derived from PR8. As far as we know, this is the first large scale sequence analysis of HYR candidate virus seeds used in seasonal and pandemic vaccine production over decades.

Our laboratory at New York Medical College (NYMC) specializes in producing HYR seed viruses for both seasonal and pandemic influenza vaccines by the classical reassortment method. Earlier, we reported the gene constellation analysis of a panel of HYRs prepared as candidate vaccine viruses and demonstrated that the gene ratio and gene constellation yielding a reassortant with hy phenotype is dependent on the individual wt virus and its interaction with the hy donor virus [36]. Also, the hy property of the reassortants generally relies on the selection of pre-existing variants with mutations favorable for improved growth *in ovo* occurring in the genes for the surface glycoproteins and/or the internal (non HA and non NA) proteins [38]. Hence, to identify the mutations associated with hy characteristics *in ovo,* the complete genomes of a total of 23 HYRs prepared as candidate vaccine seeds between 1957 and 2009, from 14 different wt viruses belonging to H1N1, 1976 H1N1-SOIV, 2009 H1N1pdm, H2N2 and H3N2 subtypes were sequenced and analyzed. The mutations observed in this analysis will be of considerable interest to influenza vaccine researchers and can be applied to modification of the sequences of the wt virus and/or the hy donor virus, PR8, during the development of influenza A vaccine candidate viruses with improved growth properties *in ovo.*

## Materials and Methods

### HYRs, hy Donor, and wt Parent Viruses

HYRs designated with an X, for example, X-31b were developed by E.D. Kilbourne and his associates; HYRs designated as NYMC X- (for example, NYMC X-163, are from the laboratory of D. Bucher (Table 1). The reassortants developed at NYMC in the Bucher laboratory are called High Yield Reassortants (HYR) continuing the original designation from E.D. Kilbourne who developed the classical reassortment method [14]. In the text all HYRs are mentioned as starting with X-.

**Table 1:** Influenza A virus HYRs analyzed in this study.

HYRS	WT Virus	WT abbreviation in text
**H1N1 (X-31b-hy donor for X-113 and X-127; NYMC X-157-hy donor for X-163’s)**		
X-113^a^	A/Texas/36/1991	TX/91
X-127a,b	A/Beijing/262/1995	BJ/95
NYMC X-163	A/St. Petersburg/8/2006	SP/06
NYMC X-163A	A/St. Petersburg/8/2006	SP/06
NYMC X-163B	A/St. Petersburg/8/2006	SP/06
**H1N1-SOIV** c **(PR8-hy donor X-53′s; NYMC X-157-hy donor for X-179’s)**		
X-53a,d	A/New Jersey/11/1976	NJ/76
X-53aa,d	A/New Jersey/11/1976	NJ/76
NYMC X-179	A/California/07/2009	CA/2009
NYMC X-179A^d^	A/California/07/2009	CA/2009
**H2N2 (PR8-hy donor)**		
X-135a,e	A/Japan/305/1957	JP/57
PR8 x A/Korea/426/68^a^	A/Korea/426/1968	KO/68
**H3N2 (PR8-hy donor)**		
X-31ba,b	A/Aichi/2/1968	AC/68
X-117a,b	A/Beijing/32/1992	BJ/92
NYMC X-149C6	A/Wyoming/03/2003	WY/03
NYMC X-157^b^	A/New York/55/2004	NY/04
NYMC X-157A	A/New York/55/2004	NY/04
NYMC X-157B	A/New York/55/2004	NY/04
NYMC X-161^b^	A/Wisconsin/67/2005	WI/05
NYMC X-161B^b^	A/Wisconsin/67/2005	WI/05
NYMC X-171	A/Brisbane/10/2007	BR/07
NYMC X-171A	A/Brisbane/10/2007	BR/07
NYMC X-171B	A/Brisbane/10/2007	BR/07
NYMC X-175C^b^	A/Uruguay/716/2007	UY/07

a HYRs prepared in E.D. Kilbourne laboratory, others at Bucher laboratory at NYMC.

b Used in seasonal influenza vaccine production.

c SOIV- swine origin influenza virus.

d X-53 and X-53a were used in the 1976 swine influenza vaccine production and X-179A was used in the 2009 H1N1pdm vaccine production.

e X-135 was developed as a pre-pandemic vaccine seed candidate in the event of a reappearance of an H2N2 subtype in the population.

The hy donor virus PR8 (H1N1 subtype) was used to generate H3N2 HYRs. To develop the H1N1 HYRs, H3N2 hy reassortants were used to have a clear antigenic distinction between wt (H1N1) and donor surface glycoproteins (H3N2) to facilitate antibody selection during reassortment. Hy H3N2 donor viruses included X-31b (H3N2 HYR made between A/Aichi/2/1968xPR8 with 6 ‘internal’ genes from PR8 and the HA and NA from A/Aichi/2/1968) and NYMC X-157 (H3N2 HYR made between A/New York/55/2004xPR8 with HA and NA from A/New York/55/2004; X-157 was subsequently found to be a mixture of 6∶2 and 5∶3 viruses with PB1s derived from both parents). In the case of the 1976 swine influenza vaccine, PR8 was used to generate the 1976 H1N1-SOIV reassortants X-53 and X-53a.

The HYRs with more than one in the same series (for example, X-163 series, X-171 series etc) are derived from independent co-infection experiments performed in parallel using the same wt viruses. A total of 38 viruses including 23 HYRs, 14 wt viruses and the hy donor virus PR8 were sequenced and analyzed.

### Viral RNA Isolation and RT-PCR Amplification of M Gene

The quality of the extracted RNA for sequencing purposes was checked by amplifying the M gene of all 38 samples by RT-PCR. Viral RNA was isolated from 280 µl of allantoic fluid using QIAmp® Viral RNA Mini Kit (Qiagen Inc., Valencia, CA) per the manufacturer’s recommendations. RNA preparations were stored at −20°C until further use. RT-PCR was carried out using Takara One Step RNA PCR Kit (Clontech, Mountain View, CA) according to the manufacturer’s recommendations. The RT-PCR reaction mixture and the thermal conditions used were as previously published [36]. The reactions were performed on an Eppendorf Mastercycler®. The amplified RT-PCR products were visualized on a 2% agarose gel with ethidium bromide in Tris Acetate EDTA buffer.

### Influenza A Virus HYR Genome Sequencing

The complete genome sequencing of the 38 RNA preparations was carried out at J. Craig Venter Institute (JCVI, Maryland, USA) under the NIAID Influenza Genome Sequencing Project (NIGSP). Whole-genome sequencing of isolates was performed using the high-throughput sequencing pipeline. The protocol was as follows. Briefly described, an M13 sequence tag was added to the 5′ end of each degenerate primer used for RT-PCR. Primers were designed to produce approximately 500-nucleotide (nt) overlapping amplicons and provide 2X coverage of each genomic segment. Each amplicon pair overlaps with its neighboring amplicon by approximately 100 nt. Additionally a second set of primers was designed to produce 500-nt amplicons offset by about 250 nt from the original primer pair, providing at least 4X sequence coverage of each segment. Primers were arranged in a 96-well plate format; all RT-PCRs for each sample were performed in one plate. Genomic RNA was amplified directly by RT-PCR and sequenced. Ninety-six RT-PCRs reactions were performed per RNA sample using a One-Step RT-PCR kit (Qiagen Inc., Valencia, CA). Sequencing reactions were performed using Big Dye Terminator chemistry (Applied Biosystems, Carlsbad, CA) with 2 µl of template cDNA. Each amplicon was sequenced from each end using M13 primers, and sequencing reactions were analyzed on a 3730 ABI sequencer (Applied Biosystems, Carlsbad, CA).

### Genome Analysis

The sequences as determined for our laboratory PR8 internal gene sequences had nucleotides missing at both ends. Therefore, the PR8 gene sequences were compared with the PR8 sequences from Schickli et al [39], to obtain the numbering for the full length gene segments to permit alignment and analysis. H1 numbering is used for the changes in HYRs deriving the internal genes from PR8 [39]. The numbering for changes in the HYR’s HA, NA and the internal genes derived from the wt viruses are according to the subtype of the wt viruses [29], [40]. The sequences were aligned using the CLUSTAL W algorithm [41]. The nucleotide and amino acid (aa) substitutions and sequence homology were determined and analyzed using the MegAlign® module of the DNASTAR package (DNASTAR, Inc., Madison, USA).

### GenBank Accession Numbers

The complete genome sequence of all 23 HYRs, 14 wt viruses and PR8 were submitted to GenBank via JCVI. The GenBank accession numbers of influenza A wt viruses and HYRs analyzed in this study are given as supporting information (Table S1). The sequences for the HA and NA of wt virus AC/68 (HA gene: AB295605; NA gene: AB295606) and wt virus UY/07 (HA gene: EU716426 and NA gene: EU716427) were used from publicly available GenBank sequences. The PB1, HA and NA sequences of 2009 H1N1pdm wt virus CA/2009 were used from published data [42].

### Statistical Analysis

Statistical significance of the nucleotide changes in terms of fold increase in HA titer was analyzed using Student’s t-test. A value of p<0.05 was considered statistically significant.

## Results

### Gene Constellation of HYRs

**Figure 1:**
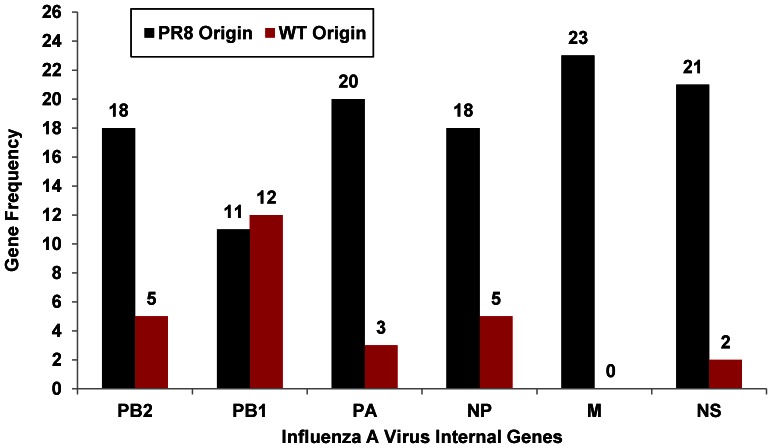
Frequency of origin of ‘internal’ genes in HYRs in the present study.

**Figure 2:**
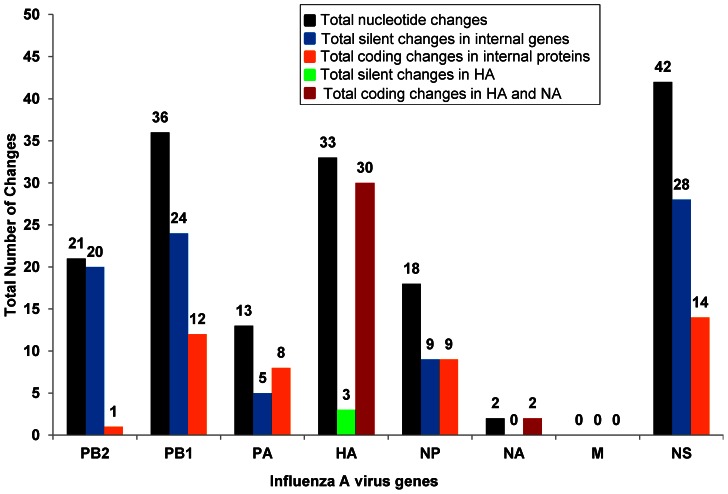
Total number of nucleotide changes in HYRs. Total Changes: The majority of the nucleotide changes (Silent and Coding, black bars) in the internal genes was found in the NS gene followed by PB1, PB2, NP, and PA. The HA gene had a total of 33 nucleotide changes; in contrast NA had only 2 nucleotide changes, both coding changes. M gene had no nucleotide change in any of the 23 HYRs. Silent Changes: Silent nucleotide changes were found in the internal genes of the HYR derived from hy donor virus, PR8 (blue bars). No silent changes were found in the internal genes derived from wt viruses except in NYMC X-157 PB1 derived from wt virus NY/04 which had 13 silent changes. HA had 3 silent nucleotide changes (green bar). NA and M genes had no silent changes in any of the 23 HYRs. Coding Changes: Among the internal proteins, the majority of the coding changes (orange bars) were found in PR8 derived NS (14 total; 7 in NS1 and 7 in NS2), followed by PB1 (10 total; 7 in PB1 and 3 in PB1-F2), NP (9) and PA (8). PB2 had only 1 coding change in contrast with 20 silent changes. M had no coding changes in any of the 23 HYRs. HA had 30 coding changes; in contrast NA had only 2 coding changes (red bars).

**Figure 3:**
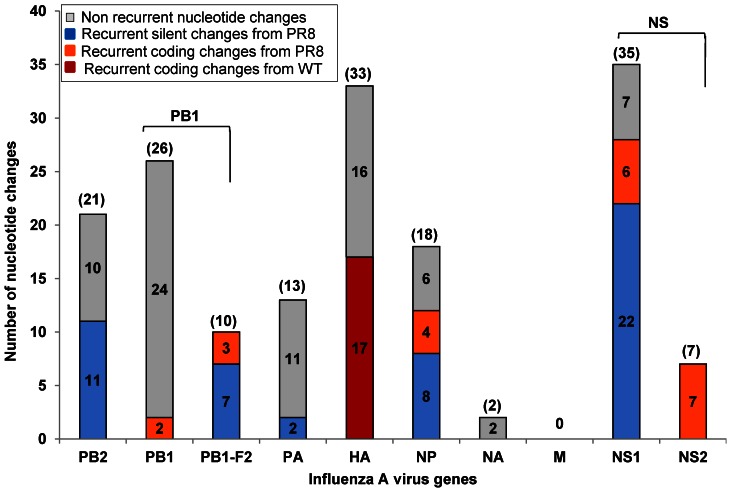
Total number of recurrent silent and recurrent coding changes in HYRs. The majority of PR8 derived recurrent silent changes are found in NS1 (22) followed by PB2, NP, PB1-F2, and PA. The majority of the PR8 derived recurrent coding changes are found in NS2 followed by NS1, NP, PB1-F2 and PB1. HA had a total of 17 recurrent coding changes involving more than one amino acid position (Table 9). NA and M genes had no recurrent changes.

All HYRs mentioned in this study were generated by classical reassortment *in ovo.* The frequency of origin of ‘internal’ genes in the HYRs is shown in Fig. 1. Apart from PR8 internal genes, all the H3N2 internal gene segments (except H3N2 M gene) were incorporated at least once among the 23 HYRs. Among the ‘desired’ gene constellations associated with high growth (6∶2 or 5∶3), 43% of the vaccine seeds (10/23) contained 6∶2 and 27% (6/23) contained 5∶3 gene compositions. Three HYRs had 4∶4; two had 3∶5; one had 2∶6 and one had 1∶7 gene compositions. Thirteen vaccine seeds acquired the complete polymerase complex from the same parental virus (PR8-44% and wt-13%). 18/23 HYRs (78%) derived both PB2 and PA from PR8. Among the wt derived genes, the wt NP was accompanied with at least one of the polymerase subunits from wt virus in 5/23 (22%). The gene compositions and the number of mutations is given in Table 2. The silent and coding changes in H1N1, 1976 H1N1-SOIV, 2009 H1N1pdm and H2N2 HYRs are given in Table 3 and Fig. 2. Silent and coding changes in H3N2 HYRs are presented in Table 4 and Fig. 2. The number of recurrent silent and coding changes per gene per parental origin is shown in Fig. 3.

**Table 2:** Gene composition and number of silent and coding changes per gene in HYRs.

Subtype	HYRs	Gene Composition							
	(Gene Ratio)	No. of Silent Changes (No. of Coding Changes)							
	(PR8:WT)	PB2	PB1	PA	HA	NP	NA	M	NS
**H1N1**									
	X-113 (4∶4)	*0* a	**0(1)** b	**0**	*0(1)*	*0(2)*	*0*	**0**	**2(2)**
	X-127 (5∶3)	**1**	*0*	**0**	*0(2)*	**0**	*0*	**0**	**2(2)**
	X-163 (6∶2)	**0**	**0(2)** c	**0(1)**	*0*	**0(1)**	*0*	**0**	**0**
	X-163A (6∶2)	**0**	**0(1)** c	**0**	*0*	**0(1)**	*0*	**0**	**0**
	X-163B (6∶2)	**0**	**1(1)** c	**0**	*0*	**0(1)**	*0*	**0**	**0**
**H1N1-SOIV/2009 H1N1pdm**									
	X-53 (5∶3)	**1**	*0* d	**1**	*1(2)*	**0**	*0*	**0**	**1**
	X-53a (5∶3)	**1**	*0(1)*	**2**	*0(1)*	**0**	*0*	**0**	**1**
	X-179 (5∶3)	**0**	*0*	**0(2)**	*0(4)*	**0**	*0*	**0**	**1**
	X-179A (5∶3)	**0**	*0*	**0**	*0(3)*	**0(1)**	*0*	**0**	**4**
**H2N2**									
	X-135 (4∶4)	**1**	*0*	**0(1)**	*0(1)*	*0*	*0*	**0**	**3(2)**
	PR8xKO/68 (3∶5)	*0*	*0*	**1**	*0(1)*	*0(1)*	*0*	**0**	**0**
**H3N2**									
	X-31b (6∶2)	**1**	**0**	**0**	*0(2)*	**0**	*0*	**0**	**2(2)**
	X-117 (6∶2)	**3**	**0(1)**	**0**	*0*	**3**	*0*	**0**	**2(1)**
	X-149C6 (3∶5)	*0*	*0*	*0*	*0*	**3**	*0*	**0**	**2(2)**
	X-157 (6∶2/5∶3)^e^	**1**	**A**/*A*	**0**	*0(1)*	**0**	*0*	**0**	**1**
	X-157A (5∶3)	**2**	*0*	**1**	*0(1)*	**0**	*0*	**0**	**1**
	X-157B (6∶2)	**1(1)**	**1**	**0(1)**	*0(2)*	**3**	*0*	**0**	**1**
	X-161 (1∶7)	*0*	*0*	*0*	*0*	*0*	*0*	**0**	*0*
	X-161B (6∶2)	**0**	**0(1)**	**0**	*0*	**0**	*0*	**0**	**1**
	X-171 (2∶6)	*0*	*0*	*0*	*0(3)*	*0(1)*	*0*	**0**	**2(3)**
	X-171A (4∶4)	**2**	*0*	**0**	*2(2)*	**0**	*0(1)*	**0**	*0*
	X-171B (6∶2)	**6**	**1(1)**	**0**	*0(3)*	**0**	*0*	**0**	**1**
	X-175C (6∶2)	**0**	**0(1)**	**0(3)**	*0(1)*	**0(1)**	*0(1)*	**0**	**1**
	Total genes (242)	29	33	27	37	29	37	24	26

a Genes derived from respective wt viruses are given in italics.

b Black bold font are genes derived from the hy donor virus PR8.

c Includes the coding change Cys42Phe in the PB1-F2 of the X-163 series of HYRs.

d RT-PCR/RFLP analysis showed X-53 PB1 derived from NJ/76 [36], however sequence data showed X-53 PB1 is a mixture of both PR8 and NJ/76 PB1 genes and also had more numbers of ambiguities, hence not included in the analysis.

e NYMC X-157 PB1was a mixture of both PR8 and NY/04 PB1 genes.

**A**/*A*- PB1 is a mixture of PR8 and NY/04; **A** (PR8 PB1) had only one silent change, *A* (NY/04 PB1) had 13 silent and 2 coding changes.

**Table 3:** Total nucleotide changes in H1N1 and H2N2 HYRs.

HYRs	Gene	Observed Nucleotide Changes
**H1N1**		
**X-113**	PB1	**A155G(N52S)**
	HA	**G716A(G239D)**
	NP	**C104A(P35Q), C971A(T324N)**
	NS	C147T, G549A**; G163A(E55K), G77A(G26E)**
**X-127**	PB2	A49C
	HA	**A604G(R202G), A608T(D203V)**
	NS	C147T, G549A**; G163A(E55K), G77A(G26E)**
**X-163**	PB1	**G2200A(A734T)**
	PB1-F2	**G125T(C42F)**
	PA	**G1213A(E405K)**
	NP	**A393G(I131M)**
**X-163A**	PB1-F2	**G125T(C42F)**
	NP	**A393G(I131M)**
**X-163B**	PB1	A1968G
	PB1-F2	**G125T(C42F)**
	NP	**A393G(I131M)**
**H1N1-SOIV/2009 H1N1pdm**		
**X-53**	PB2	A49C
	PA	A1410G
	HA	G525A; **G457A(E153K), G534A(A178T)**
	NS	T144C
**X-53a**	PB2	A49C
	PB1	**C1726T(H576Y)**
	PA	A1410G, G2052T
	HA	**G464A(G155E)**
	NS	T144C
**X-179**	PA	**A1691T(Q564L), G1861A(E621K)**
	HA	**G464A(G155E), A626C(K209T), G/D222D, A668G(Q223R)**
	NS	C154T
**X-179A**	HA	**A626C(K209T), G/D222D, A668G(Q223R)**
	NP	**A393G(I131M)**
	NS	G312C, G338C; A359G, G394A
**H2N2**		
**X-135**	PB2	C1540A
	PA	**A1732G(N578D)**
	HA	**G1203T(K401N)**
	NS	A123G, C147T, G549A; **G163A(E55K), G77A(G26E)**
**PR8xKO/68**	PA	A1218T
	HA	**A644C(K209T)**
	NP	**A227G(Y76C)**

Silent changes are in regular font; Coding changes are in bold font with corresponding aa changes in the parentheses.

**Table 4:** Total nucleotide changes in H3N2 HYRs.

HYRs	Gene	Observed Nucleotide Changes
**X-31b**	PB2	A49C
	HA	**G1460C(R487T), T1529G(V510G)**
	NS	C147T, G549A; **G163A(E55K), G77A(G26E)**
**X-117**	PB2	A49C, G1056A, A1953G
	PB1	**G175A(E59K)**
	NP	C369T, C426T, G924A
	NS	C147T**,** G549A; **G77A(G26E)**
**X-149C6**	NP	G210A, C369T, G924A
	NS	C147T, G549A; **G163A(E55K), G77A(G26E)**
**X-157**	PB2	A49C
	PB1^a^	G243T
	PB1^b^	A600G, T675C, T726C, C837T, A861G, T912A, T1728C,
		G1731A, C1833T, A1869G, C1902T, G1959A, A1977G;
		**C1750A(L584I), A1886G(Q629R)**
	HA	**C656A(S219Y)**
	NS	T144C
**X-157A**	PB2	C1827T, A2025G
	PA	G1504A
	HA	**C656A(S219Y)**
	NS	T144C
**X-157B**	PB2	C1068A; **C361T(P121S)**
	PB1	T2058C
	PA	**G991A(V334I)**
	HA	**G196T(V66F), C656A(S219Y)**
	NP	G210A, C369T, G924A
	NS	T144C
**X-161B**	PB1	**G2068A(V690I)**
	NS	T144C
**X-171**	HA	**G557T(G186V), 594GACdeletion(A198), T677G(I226S)**
	NP	**G349A(G117R)**
	NS	C147T, G549A; **G163A(E55K), C229T(L77F), G77A(G26E)**
**X-171A**	PB2	C1827T, A2025G
	HA	C54T, A198G; **T581C(L194P), G1465A(D489N)**
	NA	**A91G(T31A)**
**X-171B**	PB2	G594A, G1128A, C1827T, G1950A, C2073T, G2307A
	PB1	C400A; **G2068A(V690I)**
	HA	**G557T(G186V), 594GACdeletion(A198), C656A(S219Y)**
	NS	T144C
**X-175C**	PB1	**G1030A(V344I)**
	PA	**C599T(R200C), A905G(D302G), A1157G(K386R)**
	HA	**A424G(R142G)**
	NP	**A136G(K46E)**
	NA	**C95T(T32I)**
	NS	T144C

Silent changes are in regular font; Coding changes are in bold font with corresponding aa.

changes in the parentheses.

X-157 was a mixture of two populations of HYRs, one with ^a^ PR8 PB1 and a second with ^b^ NY/04 PB1.

### H1N1 HYRs ‘Internal’ Gene Sequences

H1N1 HYRs were generated using an H3N2 hy donor virus, either X-31b or X-157 for delivery of PR8 internal genes during reassortment of HYRs (Table 1). (Note: Although the original analysis of X-157 gene composition by RT-PCR/RFLP analysis showed that all six internal genes were derived from PR8, subsequent sequence analysis at JCVI found that X-157 was a mixture of two populations of HYRs, one with PR8 PB1 and a second with NY/04 PB1. However all reassortants made with the X-157 donor received PR8 PB1; none obtained the NY/04 PB1 gene).

#### PB2 gene

Among the five H1N1 HYRs, four derived the PB2 gene from PR8 of which only X-127 had a single silent nt change, A49C. X-113 derived PB2 from its TX/91 parent and had no change.

#### PB1 gene

Four of the five H1N1 HYRs derived the PB1 gene from PR8. X-163B had a silent nt change, A1968G. Coding changes were seen in X-113, Asn52Ser and in X-163, Ala734Thr. X-127 derived PB1 from its parent, BJ/95, and had no changes.

#### PA gene

All five HYRs derived PA from PR8. A single coding change Glu405Lys was found only in X-163.

#### NP gene

Four out of five of the H1N1 HYRs derived the NP gene from PR8. The three X-163 HYRs had a recurrent coding change, Ile131Met; X-127 had no change. X-113 deriving the NP gene from its TX/91 parent had two coding changes, Pro35Gln and Thr324Asn.

#### M gene

All five H1N1 HYRs derived the M gene from PR8 and had no changes.

#### NS gene

All five HYRs derived the NS gene from PR8. Both X-113 and X-127 had recurrent silent changes, C147T and G549A and a recurrent coding change Glu55Lys in NS1 and a recurrent coding change Gly26Glu in NS2. No changes to NS gene were seen in the X-163 series.

### H1N1 Swine Origin HYRs ‘Internal’ Gene Sequences

All four swine origin H1N1-SOIV HYRs had 5∶3 gene compositions with PB1, HA and NA from wt virus. X-53 and X-53a derived the remaining five genes from PR8 and the 2009 H1N1pdm reassortants X-179 and X-179A derived the remaining five genes from X-157.

#### PB2 gene

All four HYRs had PR8 PB2. Both X-53 and X-53a had the same silent nt change, A49C; no silent changes were seen in X-179 or X-179A. No coding changes were found in the PB2 gene for any of the four SOIV HYRs.

#### PB1 gene

All four H1N1-SOIV HYRs had wt PB1. X-53a had a coding change, His576Tyr.

#### PA gene

All four HYRs had PR8 PA. Both X-53 and X-53a had the same silent change, A1410G, with X-53a containing an additional silent change, G2052T. For the two vaccine seeds (X-179 and X-179A) developed for 2009 H1N1pdm, no silent changes were seen; X-179 had two coding changes in PA, Gln564Leu and Glu621Lys.

#### NP gene

All four HYRs obtained the PR8 NP gene. X-179A had the coding change, Ile131Met; this change was also found in the H1N1 X-163 series (Table 3).

#### M gene

No nt changes were seen in the PR8 derived M gene for any of the four H1N1-SOIV HYRs.

#### NS gene

All four HYRs derived the NS gene from PR8. Both X-53 and X-53a had the recurrent silent change, T144C in NS1 (Table 5 and 6). X-179 had a silent change C154T and X-179A had four silent changes, G312C, G338C, A359G and G394A in NS1.

**Table 5:** Recurrent silent nucleotide changes in HYRs.

Gene	Change	No. of HYRs	Recurrent silent changes (all PR8 origin)
PB2	A49C	6	X-31b, X-53, X-53a, X-117, X-127, X-157
	C1827T	3	X-157A, X-171A, X-171B
	A2025G	2	X-157A, X-171A
PB1-F2	A57G	7	X-31b, X-113, X-157, X-157B, X-161B, X-171B, X-175C
PA	A1410G	2	X-53, X-53a
NP	G210A	2	X-149C6, X-157B
	C369T	3	X-117, X-149C6, X-157B
	G924A	3	X-117, X-149C6, X-157B
NS1	T144C	8	X-53, X-53a, X-157, X-157A, X-157B, X-161B, X-171B, X-175C
	C147T	7	X-31b, X-113, X-117, X-127, X-135, X-149C6, X-171
	G549A	7	X-31b, X-113, X-117, X-127, X-135, X-149C6, X-171

**Table 6:** HYRs internal genes with recurrent silent changes.

Gene	PB2			PB1-F2	PA	NP			NS1		
Position	49	1827	2025	57	1410	210	369	924	144	147	549
**PR8[nt]**	**AGA**	**TTC**	**ACA**	**CAA**	**GGG**	**CGG**	**CTC**	**AGA**	**AGT**	**ACC**	**GGG**
**H1N1 HYRs**											
X-113	AGA	TTC	ACA	**CAG**	GGG	CGG	CTC	AGA	AGT	**ACT**	**GGA**
X-127	**CGA**	TTC	ACA	CAA	GGG	CGG	CTC	AGA	AGT	**ACT**	**GGA**
**H1N1-SOIV HYRs**											
X-53	**CGA**	TTC	ACA	CAA	**GGA**	CGG	CTC	AGA	**AGC**	ACC	GGG
X-53a	**CGA**	TTC	ACA	CAA	**GGA**	CGG	CTC	AGA	**AGC**	ACC	GGG
**H2N2 HYRs**											
X-135	**CGA**	TTC	ACA	CAA	GGG	CGG	CTC	AGA	AGT	**ACT**	**GGA**
**H3N2 HYRs**											
X-31b	**CGA**	TTC	ACA	**CAG**	GGG	CGG	CTC	AGA	AGT	**ACT**	**GGA**
X-117	AGA	TTC	ACA	CAA	GGG	CGG	**CTT**	**AGG**	AGT	**ACT**	**GGA**
X-149C6	AGA	TTC	ACA	CAA	GGG	**CGA**	**CTT**	**AGG**	AGT	**ACT**	**GGA**
X-157	**CGA**	TTC	ACA	**CAG**	GGG	CGG	CTC	AGA	**AGC**	ACC	GGG
X-157A	AGA	**TTT**	**ACG**	CAA	GGG	CGG	CTC	AGA	**AGC**	ACC	GGG
X-157B	AGA	TTC	ACA	**CAG**	GGG	**CGA**	**CTT**	**AGG**	**AGC**	ACC	GGG
X-161B	AGA	TTC	ACA	**CAG**	GGG	CGG	CTC	AGA	**AGC**	ACC	GGG
X-171	AGA	TTC	ACA	CAA	GGG	CGG	CTC	AGA	AGT	**ACT**	**GGA**
X-171A	AGA	**TTT**	**ACG**	CAA	GGG	CGG	CTC	AGA	AGT	ACC	GGG
X-171B	AGA	**TTT**	ACA	**CAG**	GGG	CGG	CTC	AGA	**AGC**	ACC	GGG
X-175C	AGA	TTC	ACA	**CAG**	GGG	CGG	CTC	AGA	**AGC**	ACC	GGG

Recurrent silent changes are given in bold.

### H2N2 HYRs ‘Internal’ Gene Sequences

Two H2N2 HYRs were developed using hy donor virus PR8 (Table 1). PR8xKO/68 is a 3∶5 HYR deriving PA, M and NS genes from PR8 with the remaining five genes from wt virus KO/68. X-135 is a 4∶4 HYR deriving PB2, PA, M and NS genes from PR8 with the remaining 4 genes from wt virus JP/57.

#### PB2 gene

X-135 had a silent change C1540A in PR8 PB2 gene. No change was seen in wt PB2 gene of PR8xKO/68 HYR.

#### PB1 gene

Both H2N2 HYRs derived wt PB1 and had no change.

#### PA gene

Both H2N2 HYRs derived PR8 PA; PR8xKO/68 HYR had a silent change A1218T; X-135 had a coding change, Asn578Asp.

#### NP gene

Both H2N2 HYRs had wt NP gene; only PR8xKO/68 had a coding change, Tyr76Cys.

#### M gene

No silent or coding changes were found in M genes for either HYR.

#### NS gene

Both HYRs derived PR8 NS. X-135 had three silent changes A123G, C147T and G549A and a coding change Glu55Lys in NS1. In addition X-135 also had a coding change Gly26Glu in NS2. PR8xKO/68 had no changes.

### H3N2 HYRs ‘Internal’ Gene Sequences

Twelve H3N2 HYRs were sequenced and compared with parental sequences; all were generated using the H1N1 hy donor virus PR8 (Table 1).

#### PB2 gene

Nine H3N2 HYRs obtained the PB2 gene from PR8. Of which, seven HYRs had silent changes, including a recurrent silent change, A49C in X-31b, X-117 and X-157; C1827T in X-157A, X171A, X-171B, and A2025G in X-157A and X171A. Non-recurrent changes include, G594A, G1128A, G1950A, C2073T and G2307A (X-171B), G1056A and A1953G (X-117), C1068A (X-157B) (Table 4). A coding change was found in the PB2 gene for X-157B, Pro121Ser. No changes were seen in the three HYRs with wt PB2, X-149C6, X-161 and X-171.

#### PB1 gene

The PR8 derived PB1 for X-157 had a single silent change G243T, while the PB1 gene derived from wt virus (NY/04) for X-157 was found to have 13 silent changes (Table 4). Six H3N2 HYRs obtained the PR8 PB1. Silent changes were seen in X-157B, (T2058C) and in X-171B (C400A). Four HYRs had coding changes including, Glu59Lys in X-117, Val690Ile in X-161B and X-171B and Val344Ile in X-175C. Two coding changes, Leu584Ile and Gln629Arg were found in the WT derived X-157 PB1 gene. No changes were found in the remaining five HYRs with wt PB1.

#### PA gene

Nine of the 12 H3N2 HYRs had the PR8 PA gene. A silent change, G1504A was seen in the PR8 PA gene of X-157A. A total of four coding changes were found in the PR8 PA; one in X-157B (Val334Ile) and three in X-175C (Arg200Cys, Asp302Gly and Lys386Arg). The remaining HYRs had no changes in the PA gene regardless of parental origin (Table 2).

#### NP gene

Ten HYRs obtained the PR8 NP gene. Three HYRs with PR8 NP had silent changes, X-149C6 and X-157B had the same changes, G210A, C369T and G924A. X-117 had three silent changes, C369T, C426T and G924A. These recurrent silent changes are not found in HYRs of other subtypes. Coding changes, Gly117Arg and Lys46Glu, were seen in X-171 and X-175C, respectively. No changes were found in seven H3N2 HYRs whether the NP gene was derived from PR8 or wt.

#### M gene

No silent or coding changes were found for the PR8 derived M gene in any of the 12 H3N2 HYRs.

#### NS gene

Ten H3N2 HYRs contained the PR8 NS gene and each had at least one change. Six HYRs, X-157, X-157A, X-157B, X-161B, X-171B and X-175C had a recurrent silent change, T144C in NS1. Another group of four HYRs, X-31b, X-117, X-149C6 and X-171 had recurrent silent changes C147T and G549A in NS1 and a recurrent coding change Gly26Glu in NS2 (Table 7 and 8). From this group, except X-117, the other three HYRs, X-31b, X-149C6 and X-171 had a recurrent coding change, Glu55Lys in NS1. In addition, X-171 had another coding change Leu77Phe in NS1. No changes were seen in the two HYRs with the wt NS gene.

**Table 7:** Recurrent Coding Changes in HYRs.

Protein	Change	No. of HYRs	Recurrent coding changes
PB1	V690I	2	X-161B, X-171B
PB1-F2	C42F	3	X-163, X-163A, X-163B
NP	I131M	4	X-163, X-163A, X-163B, X-179A
NS1	E55K	6	X-31b, X-113, X-127, X-135, X-149C6, X-171
NS2	G26E	7	X-31b, X-113, X-117, X-127, X-135, X-149C6, X-171
HA	G155E	2	X-53a, X-179
	G186V	2	X-171, X-171B
	A198deletion	2	X-171, X-171B
	K209T	3	X-179, X-179A, PR8xA/KO/68
	S219Y	4	X-157, X-157A, X-157B, X-171B
	G/D222D	2	X-179, X-179A
	Q223R	2	X-179, X-179A

Single letter amino acid code is used.

The recurrent coding changes were found in the ‘internal’ genes of PR8 origin and in the HA genes from the respective wt viruses.

**Table 8:** HYR internal genes with recurrent coding changes.

Proteins	PB1	PB1-F2	NP	NS1	NS2
**nt[aa]**	2070(690)	125(42)	393(131)	163(55)	77(26)
**PR8 nt[aa]**	**GTA(V)**	**TGC(C)**	**ATA(I)**	**GAG(E)**	**GGG(G)**
**H1N1**					
X-113	GTA(V)	TGC(C)	ATA(I)	**AAG(K)**	**GAG(E)**
X-127	GTA(V)	TGC(C)	ATA(I)	**AAG(K)**	**GAG(E)**
X-163	GTA(V)	**TTC(F)**	**ATG(M)**	GAG(E)	GGG(G)
X-163A	GTA(V)	**TTC(F)**	**ATG(M)**	GAG(E)	GGG(G)
X-163B	GTA(V)	**TTC(F)**	**ATG(M)**	GAG(E)	GGG(G)
**H1N1-SOIV**					
X-179A	GTA(V)	TGC(C)	**ATG(M)**	GAG(E)	GGG(G)
**H2N2**					
X-135	GTA(V)	TGC(C)	ATA(I)	**AAG(K)**	**GAG(E)**
**H3N2**					
X-31b	GTA(V)	TGC(C)	ATA(I)	**AAG(K)**	**GAG(E)**
X-117	GTA(V)	TGC(C)	ATA(I)	GAG(E)	**GAG(E)**
X-149C6	GTA(V)	TGC(C)	ATA(I)	**AAG(K)**	**GAG(E)**
X-161B	**ATA(I)**	TGC(C)	ATA(I)	GAG(E)	GGG(G)
X-171	GTA(V)	TGC(C)	ATA(I)	**AAG(K)**	**GAG(E)**
X-171B	**ATA(I)**	TGC(C)	ATA(I)	GAG(E)	GGG(G)

Single letter amino acid code is used.

The recurrent coding changes were found in the ‘internal’ genes of PR8 origin and are given in bold.

### PB1-F2 Region of PB1

A total of 10 HYRs derived PB1 from PR8 and retained the 87 aa long PB1-F2. The HIN1 HYR, X-127 with BJ/95 PB1 had a truncated 57aa long PB1-F2. The H1N1 SOIV HYRs, X-53a had truncated 52 aa long PB1-F2; X-179 and X-179A had a truncated PB1-F2 at 12 aa. The H2N2 HYRs (X-135 and PR8xKO/68) and five out of 12 H3N2 HYRs (X-149C6, X-157A, X-161, X-171 and X-171A) with wt derived PB1 had a PB1-F2 protein size of 90 aa. X-157 which was a mixture had PB1 from both PR8 and wt viruses (see earlier comment, see Note: Section B) had PB1-F2 proteins of 87 and 90 aa, respectively. The HYRs deriving PB1 (encoding PB1-F2) from PR8 (except X-117 and X-163 series) had a single recurrent silent nt change, A57G. Whereas, the X-163 series of HYRs had a recurrent coding change, Cys42Phe, which is the only coding change in the PB1 gene associated with PB1-F2.

### HA Gene Sequences

The HYR seed viruses must derive HA and NA genes from their respective wt viruses. Among the HYRs analyzed, three silent changes and 30 coding changes were found in the HA genes (Tables 2, 3 and 4).

#### H1N1

A silent nt change was found in X-53 (G525A). X-53 and X-127, each had two coding changes in the HA; Glu153Lys and Ala178Thr (X-53), and Arg202Gly and Asp203Val (X-127). X-113 had a single coding change, Gly239Asp. X-53a had a coding change, Gly155Glu which was also found in X-179 (a 2009 H1N1pdm HYR). In addition, X-179 and X-179A shared three coding changes, Lys209Thr, Gly/Asp222Asp and Gln223Arg.

#### H2N2

The Lys209Thr change seen in X-179 and X-179A was also found in an H2N2 HYR (PR8xKO/68) (Table 9).

**Table 9:** HYR hemagglutinins with recurrent coding changes.

Coding Changes at Positions^a^						
G/D222D^b^	G155E	G186V	K209T	Q223R	S219Y	A198deletion
X-179	X-53a	X-171	X-179	X-179	X-157	X-171
X-179A	X-179	X-171B	X-179A	X-179A	X-157A	X-171B
			PR8xKO/68		X-157B	
					X-171B	

a Single letter amino acid code used.

b WT parent virus CA/2009 had either G or D; the HYRs had D at position 222.

Amino acid numbering was according to HA subtypes (refer Table 1 for HYR subtype).

#### H3N2

X-171A had two silent nt changes, C54T and A198G. Coding changes were found in the X-157 series of HYRs including the same recurrent coding change, Ser219Tyr which was also found in X-171B (Table 9). X-157B had a coding change, Val66Phe. Both X-171 and X-171B had the coding change, Gly186Val, together with an alanine deletion at position 198. X-171A had a coding change, Leu194Pro. The H3N2 vaccine virus, X-175C, had a single coding change Arg142Gly.

#### HA2 region

Four coding changes were found in the HA2 region, Lys401Asn (X-135, H2N2); Arg487Thr and Val510Gly (X-31b, H3N2) and Asp489Asn (X-171A, H3N2) in the amino terminus of the HA2. These changes are unique to this study.

#### HA coding changes and structure

**Figure 4:**
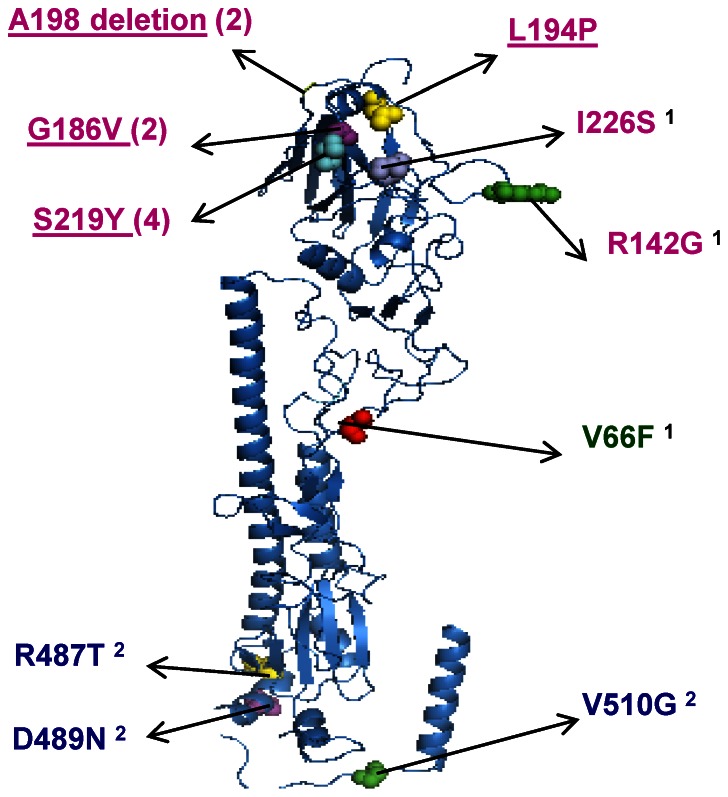
Structural model of H3N2 HA (monomer) with coding changes found in the H3N2 HYRs in this study. The structure was generated by PyMOL Molecular Graphics System (www.pymol.org). Single letter amino acid code is used. The numbers in the parentheses indicate recurrent changes (see Table 9); underlined changes were reported earlier [25]–[27], [29], [43]–[45] and also found in our study. ^1^indicates unique changes found in the HA1 in this study and ^2^ indicates unique changes found in the HA2 region.

Some of the coding changes including Gly155Glu, Gly186Val, Leu194Pro, Lys209Thr, Ser219Tyr, Gly/Asp222Asp and Gln223Arg, had been reported earlier to affect receptor specificity and egg adaptation [26]–[29], [43]–[45]. The coding changes found in the H3N2 HYRs are depicted in the structural model of an H3N2 HA generated by PyMOL Molecular Graphics System (www.pymol.org) (Fig. 4).

### NA Gene Sequences

In our study, no silent nt changes were found in the NA gene for any of the 23 HYRs. In contrast to HA with 31 coding changes, only two HYRs had coding changes in the NA genes, both were N2 subtype, Thr31Ala in X-171A (H3N2) and Thr32Ile in X-175C (H3N2). Both are located in the amino terminal end which anchors NA in the viral envelope.

### Codon Usage in HYR ‘Internal’ Genes of PR8 Origin

Both recurrent and individual silent changes were observed in all the internal genes of the HYRs derived from PR8 except the M gene. The A49C change found in the PB2 gene of six HYRs (Table 5 and 6) is at the first codon of the arginine residue. Apart from this recurrent change, the PB2 genes of five other HYRs had a total of 14 silent nt changes (five G-A, four C-T, three A-G and two C-A conversions). In the PB1 gene, the 17 silent changes included five A-G, four T-C, three C-T, two G-A, one G-T, one C-A and a T-A conversion. The seven silent nt changes in the PB1-F2 gene are A-G conversions at the third codon of a glutamine residue. In PA, four HYRs had silent changes, two A-G, followed by a single conversion of G-A, G-T or A-T. The HA gene had three silent changes (one G-A, one A-G and one C-T conversions). Three HYRs had a total of nine silent nt changes in the NP genes (five G-A and four C-T conversions). Among the HYR genes analyzed, NS1 had the maximum number of silent changes, 9 C-T, 14 G-A, 8 T-C, 2 A-G and 2 G-C conversions and NS2 had 7 G-A conversions.

### Statistical Analysis

The statistical significance of the recurrent changes was determined using Student’s ‘t’ test and found the recurrent coding changes in NS1 (p<0.0331), NS2 (p<0.0012), NP (p<0.026) and PB1-F2 (p<0.0075) (Table 7) are statistically significant as compared to the non recurrent coding changes in terms of fold increase in the HA titer.

### HYR Seed Viruses used in Vaccine Production

Out of 23 HYRs analyzed in this study, the mutations found in 9 HYR vaccine seed viruses used in vaccine preparation since X-31b are given in Table 10 alongside their gene constellation.

**Table 10:** Mutations in influenza A HYR seed viruses used in vaccine preparation.

HYRs	Gene Ratio	Genes	Nucleotide and Amino Acid Changes
**H1N1**			
**X-127** a	5∶3	PB2	A49C
1999-2000 vaccine		HA	**A604G(R202G), A608T(D203V)**
		NS	C147T, G549A**; G163A(E55K), G77A(G26E)**
**H1N1-SOIV**			
**X-53** a	5∶3	PB2	A49C
1976 Swine		PA	A1410G
Flu vaccine		HA	G525A; **G457A(E153K), G534A(A178T)**
		NS	T144C
**X-53a** a	5∶3	PB2	A49C
1976 Swine		PB1	**C1726T(H576Y)**
Flu vaccine		PA	A1410G, G2052T
		HA	**G464A(G155E)**
		NS	T144C
**X-179A** b	5∶3	HA	**A626C(K209T), G/D222D, A668G(Q223R)**
2009-2013(Present)		NP	**A393G(I131M)**
		NS	G312CA, G338C; A359G, G394A
**H3N2**			
**X-31b** a	6∶2	PB2	A49C
1971-73vaccines		HA	**G1460C(R487T), T1529G(V510G)**
		NS	C147T, G549A; **G163A(E55K), G77A(G26E)**
**X-117** a	6∶2	PB2	A49C, G1056A, A1953G
1993-94		PB1	**G175A(E59K)**
vaccine		NP	C369T, C426T, G924A
		NS	C147T**,** G549A; **G77A(G26E)**
**X-157** b	6∶2/5∶3	PB2	A49C
2005-06		PB1^c^	G243T
vaccine		PB1^d^	A600G, T675C, T726C, C837T, A861G,
			C1902T, G1959A, C1833T, A1869G, T912A,
			T1728C, G1731A, A1977G; **C1750A(L584I),**
			**A1886G(Q629R)**
		HA	**C656A(S219Y)**
		NS	T144C
**X-161B** b	6∶2	PB1	**G2068A(V690I)**
2006-08 vaccines		NS	T144C
**X-175C** b	6∶2	PB1	**G1030A(V344I)**
2008-2011		PA	**C599T(R200C), A905G(D302G), A1157G(K386R)**
vaccines		HA	**A424G(R142G)**
		NP	**A136G(K46E)**
		NA	**C95T(T32I)**
		NS	T144C

Silent changes are in regular font; Coding changes are in bold font with corresponding aa changes in the parentheses.

a HYRs prepared in E.D. Kilbourne laboratory.

b HYRs prepared in Bucher laboratory at NYMC.

X-157 was a mixture of two populations of HYRs, one with ^c^ PR8 PB1 and a second with ^d^ NY/04 PB1.

## Discussion

The first inactivated IAV vaccine was produced in chicken eggs in the 1940s using wt virus. Since clinical isolates grow to low titer in embryonated chicken eggs, influenza HYRs were developed by E.D. Kilbourne for use as the ‘seed’ virus for influenza vaccines and continue to be used for vaccine production to the present. The hy property of reassortants depends on the natural selection of variants with mutations favorable to growth. HYRs which derive PR8 ‘internal’ genes generally grow better than the original wt virus. However, the egg adaptive changes which occur in the internal genes of the HYRs are not well defined. Hence, in this study, the complete genome sequences of 23 HYRs developed from 14 different wt viruses and the hy donor PR8 were analyzed.

The polymerase complex of influenza virus is comprised of PB2, PB1 and PA. The PB2 gene is one of the major components of the replication complex. HYRs with either PR8 or wt PB2 gene retained a lysine residue at position 627 which has been associated with host adaptation and virulence [46]–[50]. It has been demonstrated that the PB2 gene plays an important role in regulating host anti-viral response by inhibiting IFN-β production [51]–[52]. A large number of silent changes (20) were seen in the PB2 gene which indicates the importance of adaptation to the egg host at the RNA level; 11/20 are recurrent changes (Table 5, 6 and Fig. 3). The silent change, A49C was found in six HYRs (X-31b, X-53, X-53a, X-117, X-127 and X-157) selected for vaccine production suggesting the possible importance of this nt change to high growth *in ovo.* The only coding change in PB2, Pro121Ser, was found in X-157B and this change may alter secondary structure.

PB1 plays a key role in cross-species infection and pathogenesis [49] and was the most frequent internal wt gene segment present in the HYRs. 12/23 HYRs derived the wt PB1 gene (Fig. 1). Ten of the 23 HYRs were used in vaccine production and 5/9 had the wt PB1 genes. The presence of the wt PB1 segment in HYRs used in vaccines including the CA/2009 PB1 in the HYRs used for the 2009 H1N1pdm production of vaccine must provide a selective advantage for growth. With regard to HYRs deriving the wt PB1 gene, X-53a had a His576Tyr coding change suggesting that this coding change may also have contributed to the higher growth of X-53a *in ovo* [44]. The 2009 H1N1pdm vaccine seed viruses X-179 and X-179A derived CA/2009 PB1 gene and had no changes, which suggests the importance of wt PB1 in these reassortants. Wanitchang and co-workers also reported that a 5∶3 reassortant with CA/2009 PB1 gene had a higher growth rate after transfection compared to that of 6∶2 reassortants with the PR8 PB1 gene [53]. Since the frequency of HYRs deriving wt PB1 gene is high; comparison of the silent and coding changes found in PR8 and wt derived PB1 may help in understanding the role of polymerase activity through the PB1 component in terms of viral growth.

PB1-F2, the second protein encoded in the PB1 gene has been implicated in pathogenicity [4], [54], [55] and has been shown to function in an anti-interferon manner like NS1 [56]. Eleven HYRs acquired the PB1 gene from PR8; seven of which had the recurrent silent change A57G in PB1-F2. The X-163 series of HYRs had a recurrent coding change Cys42Phe in PB1-F2 of the PB1 protein. The importance of these silent and coding changes in pathogenicity, including anti-interferon properties and in *in ovo* replication, has not been reported.

The PA subunit of the polymerase complex is reported to induce generalized proteolysis, an activity found in the amino-terminal third of the PA protein, 247 residues [57], [58]. The PA endonuclease activity is also associated with the amino-terminal end; the activity resides in the first 209 residues [59]. X-175C with PR8 derived PA has a coding change, Arg200Cys, in the amino-terminal region, which may affect the proteolysis and/or endonuclease activities of PA protein for better growth *in ovo.* Other individual coding changes observed in HYRs deriving PA from PR8, involving residues which may affect the structure and polarity of the PA protein may allow more efficient interaction with the other polymerase subunits. Of interest, in three H3N2 HYRs (X-149C6, X-161 and X-171), PA, PB2 and PB1 gene segments are incorporated from wt viruses. This may permit better co-operativity of the polymerase subunits for these reassortants.

Conserved regions of IAV polymerase genes critical for efficient vRNA packaging have been reported to play a role in RNA structure, RNA-RNA interactions and RNA-protein interactions [60]. Muramoto and co-workers reported that there is a strong correlation between the level of PB2 packaged and the levels of other internal gene vRNAs packaged [61]. In our study, X-157A (5∶3, with wt PB1) and X-171A (4∶4 with wt PB1 and NS) had recurrent silent changes, C1827T and A2025G in the PR8 derived PB2 genes with no change in the wt derived PB1. X-157A had a 32-fold increase in HA titer whereas X-171A had only a two-fold increase in HA titer increase compared to their respective wt viruses [36]. In these cases, in terms of growth, even though both had the same PB2 mutations, the wt derived NS gene in X-171A was not associated with enhanced growth as compared with the PR8 derived NS in X-157A which had one silent and one coding change. Thus these non-coding changes found in PB2 along with native PB1 and NS may have resulted in altered viral packaging thus resulting in differences in their HA titer.

Our analysis of HYRs also showed some correlation between coding mutations in the polymerase subunits. Although PB2, PB1 and PA interact with each other in a specific manner in controlling virus replication, the PB1-PA interaction unlike the PB2-PA interaction, has been well-characterized [17], [62]-[64]. In our study, HYRs had changes in PB1 and PA regardless of the origin of the parental genes. The H3N2 HYR, X-175C (used in 2008-09 seasonal vaccine), derived both PB1 and PA from PR8, had Val344Ile in PB1 and three coding changes Arg200Cys, Asp302Gly and Lys386Arg in PA. The role of these changes in efficient interaction of the PB1-PA is not known. X-157B had the coding changes, Pro121Ser and Val344Ile, in the PR8 derived PB2 and PA, respectively. The coding change, Pro121Ser will affect the secondary structure of PB2 while the Val334Ile change is conservative with the introduction of a slightly longer branched aa chain in PA. These changes in the structure of PB2 and PA may help the X-157B viral components interact more efficiently to improve the viral growth in comparison to its low growing wt virus.

The NP and its interaction with the polymerase subunits is well documented. Earlier studies showed that the presence of PR8 NP was associated with hy phenotype *in ovo* [30], [31], [65]. For example, X-175C had coding changes Lys46Glu and Val344Ile in PR8 derived NP and PB1, respectively. The presence of wt derived NP with or without mutations in co-operation with either PR8 or wt derived polymerase subunits in HYR growth must be considered. For example, PR8xKO/68 and X-171 had coding changes in wt NP with no change in wt derived PB1 and PB2. Replacement of the tyrosine with cysteine may affect the NP structure of PR8xKO/68 with potential formation of an additional disulfide bond. Introduction of a large basic arginine will increase the overall charge and hydrophilicity and decrease the flexibility of the peptide chain of NP for X-171. These coding changes in PR8 or wt NP suggest they may play a role in efficient interaction with the polymerase complex for better functioning of the vRNPs.

PR8 M gene is associated with the high growth phenotype *in ovo* [31], [66]–[68] and for morphology and progeny particle assembly [69]. All 23 HYRs derived the M gene from PR8 and no silent or coding changes were seen. This shows that the highly conserved M gene of PR8 origin by itself or in combination with other PR8 genes will improve reassortant virus growth. The importance of M gene in other donor viruses was also demonstrated earlier [68]. However, using WSN virus (not PR8 M gene), Klimov and co-workers reported that the presence of WSN M in the reassortants does not guarantee higher growing viruses, because the contribution of the M can be altered by properties of other genes and the proteins they encode from the parental viruses [32]. This was true with the X-161 series of HYRs. The gene constellation of X-161 is 1∶7 deriving only the PR8 M gene, and X-161B is a 6∶2 HYR deriving all the internal genes from PR8. In this case, the difference in the gene constellations plays a key role in determining growth *in ovo,* even though few coding changes were found (Table 4).

The NS gene of influenza virus encodes two distinct proteins, NS1 [1] and NS2/NEP [70]. NS1 plays a role in virulence [71], is an interferon antagonist [72]–[73], enters and accumulates in infected cells [74] and co-immunoprecipitates with NS1-specific serum along with the polymerase subunits and NP [75]. With the exception of the X-163 series and wt derived NS genes, all HYRs had either a silent or a coding change along with changes in one or more components of the vRNPs (Table 3 and 4). In NS1, the recurrent silent change T144C was found in 8; C147T in 7 and G549A in 7 HYRs (Table 5 and 6). Whereas the recurrent coding changes Glu55Lys (NS1) and Gly26Glu (NS2) was found in 6 and 7 HYRs, respectively (Table 7 and 8). These changes in NS1 and NS2 suggest the importance of PR8 derived silent and coding changes even though the HYRs were developed from wt viruses of different subtypes. The NS2/NEP protein is involved in nuclear export of nascent influenza vRNA and regulates viral transcription and replication [76]. In this analysis we found that the highest numbers of silent and coding changes were found in the NS gene, which highlights the importance of variability in the NS gene and gene products in the enhanced replication of HYRs *in ovo*.

The essential requirement for HYR vaccine seed candidates is that both the HA and NA genes must be derived from wt virus. In the HA, deletion of alanine at position 198 in X-171 and X-171B was responsible for a more restrictive receptor binding pattern [29]. Coding changes at positions 153, 155 [43]–[44], 186, 194 and 219 [29], [45], 222, 223 and 226 [25]–[27], [45] were all reported to be associated with egg adaptation. Both X-179 and X-179A had the recurrent coding changes, Lys209Thr and Gln223Arg, which were assumed to be associated with higher growth of these HYRs. The enhanced growth properties in the X-157 series may be due to the recurrent coding change Ser219Tyr which has been established as a residue associated with egg adaptation.

Changes at the antigenic sites have the potential to increase virus growth and are allowable in HYRs as vaccine seeds as long as antigenicity is not significantly altered. For example, the H3N2 vaccine component X-175C used in seasonal vaccines in 2008 through 2010 had the coding change Arg142Gly within antigenic site A with no altered antigenicity. However, changes at residues 153, 154 and 155 within the antigenic site and in the vicinity of RBS may reduce viral antigenicity [43], [44], [77]. HYRs that have changes at these residues may be excluded as vaccine candidates, even though these changes may improve virus growth. For example, even though a Gly155Glu mutation was associated with significant fold increase in HA titer over the wt virus, there was altered antigenicity in a CA/2009 reassortant candidate developed by Chen and co-workers for the live attenuated vaccine which made them unsuitable as vaccine candidates [28]. This Gly155Glu mutation was also seen in X-53a [44] and in X-179 [78]. X-179 with high growth properties had been considered a strong candidate ‘seed’ for H1N1pdm vaccine; however the Gly155Glu mutation altered the antigenicity and was not acceptable for use in the 2009 H1N1pdm vaccine. On the other hand, Gly186Val was reported to improve the growth of CA/2009 [28], A/Fujian/411/2002 [45] and A/Singapore/21/04 [79] without affecting antigenicity. Widjaja *et al.* [45] reported that adaptation of A/Fujian/411/2002 *in ovo* was due to the coding change Gly186Val, at aa position 186. At CSL, Ltd., a series of genetically modified reassortants based on the X-179A sequence data were developed [80] and were found to have improved *in ovo* growth, which further implicates the importance of these residues Lys209Thr and Gln223Arg in virus adaptation and growth. In comparison to X-179A, another H1N1pdm seed virus, X-181 had an additional coding change, Asn129Asp, which may have contributed to the higher growth of X-181 as compared with X-179A [78].

Coding changes were found in the HA2 region of the HA genes of H2N2 and H3N2 HYRs which were not previously reported and are unique to this study. These changes are found in the amino terminus region of the HA2 protein of X-135 (Lys401Asn), X-31b (Arg487Thr and Val510Gly) and X-171A (Asp489Asn). Although the number of changes are fewer than seen for HA1, it is interesting to note that three of four changes in HA2 involve loss of charged aa residues (aspartic acid, lysine and arginine) which will lead to a reduction in overall protein charge. As the amino terminus region of HA2 is involved in virus entry [40], the role of these residues in egg adaptation should be studied further.

The NA of influenza virus is a homotetramer and functions in the release of progeny virus from the infected host cell [81]–[83]. It has been reported that functional balance of HA and NA regulates the efficiency of virus replication [84]. Similar to HA, NA is also under evolutionary pressure from serum antibodies existing in the population [85]. In this study, only two H3N2 HYRs showed coding changes in the NA, Thr31Ala in X-171A and Thr32Ile in X-175C. Both coding changes were found in the hydrophobic NH_2_ terminus of NA, which is inserted in the membrane [86]. These changes may facilitate growth *in ovo*. No changes are seen at the active site of the NA in contrast to the many changes seen in the RBS in HA. Evidently, wt NA is well-suited to replicate *in ovo* or readily accommodates the changes in HA for optimal viral replication.

The importance of silent mutations on virus replication and evolution has been shown [87]–[92]. Silent mutations in RNA viruses may be far from neutral and can contribute significantly to host adaptation and become fixed in the viral RNA quasi-species populations during genetic drift [93]–[96]. A recurring silent change, A49C in PB2 and A57G in PB1-F2 are assumed to be primarily derived from PR8 variants which are positively selected by the egg host. Wong and co workers showed that silent changes in PB2 affect replication and adaptation [97]; as also seen in the present study where 20 out of 21 changes in the PB2 gene were silent. Selection pressures acting against the use of G or C at the third codon position [98] and primarily C to T mutation bias in human influenza viral genomes has been reported [17], [97], [99]. Reduced GC content in influenza viruses may prevent the activation of the human innate immune system thereby allowing more efficient viral RNA translation [100]. In this study, the *in ovo* prepared HYRs also showed a majority of replacement of third codon G and C by A and T, thereby reducing the overall GC content.

Influenza viruses undergo Darwinian selection resulting in mutants that efficiently replicate *in ovo*. When two distinct influenza viruses replicate in embryonated chicken eggs the interactions between the virus and the host cell proteins are crucial for virus replication, assembly and trafficking [101]. A crucial aspect to the vaccine manufacturing process is to obtain a reassortant with high growth *in ovo*, thus providing the maximal amount of virus for preparation of the inactivated vaccine. The gene constellation differences (PR8:wt genes) are equally important for the high growth phenotype exhibited by the HYRs *in ovo.* Apart from the gene composition, based on the analysis of the HYRs, we hypothesize that in addition to the recurrent mutations, the individual changes found in all eight genes and their encoded proteins of HYRs prepared as candidate seed viruses may augment growth by playing important roles in *in ovo* virus replication. Hence, further identification of mutations in additional HYRs as they are developed may help in the design of high growth seed viruses with improved growth *in ovo.*

## Supporting Information

Table S1 GenBank Accession number of influenza A wt viruses and HYRs analyzed in this study.
(DOCX)
