# Supplementary material for: Molecular Signature of High Yield (Growth) Influenza A Virus Reassortants Prepared as Candidate Vaccine Seeds
Source: PLoS One. 2013 Jun 11;8(6):e65955. doi: 10.1371/journal.pone.0065955 (PMC3679156; doi:10.1371/journal.pone.0065955)
Supplement: Table S1 — GenBank Accession number of influenza A wt viruses and HYRs analyzed in this study. (DOCX) [file pone.0065955.s001.docx]

**Table S1. GenBank Accession number of influenza A wt viruses and HYRs analyzed in this study**

| **GenBank Acc #** | **Wild type and HYRs details** | **Gene** | |
| --- | --- | --- | --- |
| **H1N1 High Yield Donor Virus** | | | |
| CY033584 | A/Puerto Rico/8/1934 | PB2 | |
| CY033583 | A/Puerto Rico/8/1934 | PB1 | |
| CY033582 | A/Puerto Rico/8/1934 | PA | |
| CY033580 | A/Puerto Rico/8/1934 | NP | |
| CY033578 | A/Puerto Rico/8/1934 | M | |
| CY033581 | A/Puerto Rico/8/1934 | NS | |
| **H1N1 Wild Type and HYRs** | | | |
| CY033605 | A/Texas/36/1991 | PB2 | |
| CY033655 | A/Texas/36/1991 | HA | |
| CY033601 | A/Texas/36/1991 | NP | |
| CY033600 | A/Texas/36/1991 | NA | |
| CY033654 | A/X-113 (X-31B-Texas/36/1991) | PB2 | |
| CY033599 | A/X-113 (X-31B-Texas/36/1991) | PB1 | |
| CY033598 | A/X-113 (X-31B-Texas/36/1991) | PA | |
| CY033593 | A/X-113 (X-31B-Texas/36/1991) | HA | |
| CY033596 | A/X-113 (X-31B-Texas/36/1991) | NP | |
| CY033595 | A/X-113 (X-31B-Texas/36/1991) | NA | |
| CY033594 | A/X-113 (X-31B-Texas/36/1991) | M | |
| CY033597 | A/X-113 (X-31B-Texas/36/1991) | NS | |
| CY033620 | A/Beijing/262/1995 | PB1 | |
| CY033614 | A/Beijing/262/1995 | HA | |
| CY033616 | A/Beijing/262/1995 | NA | |
| CY044316 | A/X-127 (Puerto Rico/8/1934-Beijing/262/1995) | PB2 | |
| CY044315 | A/X-127 (Puerto Rico/8/1934-Beijing/262/1995) | PB1 | |
| CY044314 | A/X-127 (Puerto Rico/8/1934-Beijing/262/1995) | PA | |
| CY044309 | A/X-127 (Puerto Rico/8/1934-Beijing/262/1995) | HA | |
| CY044312 | A/X-127 (Puerto Rico/8/1934-Beijing/262/1995) | NP | |
| CY044311 | A/X-127 (Puerto Rico/8/1934-Beijing/262/1995) | NA | |
| CY044310 | A/X-127 (Puerto Rico/8/1934-Beijing/262/1995) | M | |
| CY044313 | A/X-127 (Puerto Rico/8/1934-Beijing/262/1995) | NS | |
| CY035126 | A/St. Petersburg/8/2006 | HA | |
| CY035128 | A/St. Petersburg/8/2006 | NA | |
| CY034131 | A/NYMC X-163 (NYMC X-157-St.Petersburg/8/2006) | PB2 | |
| CY034130 | A/NYMC X-163 (NYMC X-157-St.Petersburg/8/2006) | PB1 | |
| CY034129 | A/NYMC X-163 (NYMC X-157-St.Petersburg/8/2006) | PA | |
| CY034124 | A/NYMC X-163 (NYMC X-157-St.Petersburg/8/2006) | HA | |
| CY034127 | A/NYMC X-163 (NYMC X-157-St.Petersburg/8/2006) | NP | |
| CY034126 | A/NYMC X-163 (NYMC X-157-St.Petersburg/8/2006) | NA | |
| CY034125 | A/NYMC X-163 (NYMC X-157-St.Petersburg/8/2006) | M | |
| CY036928 | A/NYMC X-163 (NYMC X-157-St.Petersburg/8/2006) | NS | |
| CY036926 | A/NYMC X-163A (NYMC X-157-St.Petersburg/8/2006) | PB2 | |
| CY036925 | A/NYMC X-163A (NYMC X-157-St.Petersburg/8/2006) | PB1 | |
| CY036924 | A/NYMC X-163A (NYMC X-157-St.Petersburg/8/2006) | PA | |
| CY036919 | A/NYMC X-163A (NYMC X-157-St.Petersburg/8/2006) | HA | |
| CY036922 | A/NYMC X-163A (NYMC X-157-St.Petersburg/8/2006) | NP | |
| CY036921 | A/NYMC X-163A (NYMC X-157-St.Petersburg/8/2006) | NA | |
| CY036920 | A/NYMC X-163A (NYMC X-157-St.Petersburg/8/2006) | M | |
| CY035123 | A/NYMC X-163A (NYMC X-157-St.Petersburg/8/2006) | NS | |
| CY035125 | A/NYMC X-163B (NYMC X-157-St.Petersburg/8/2006) | PB2 | |
| CY035124 | A/NYMC X-163B (NYMC X-157-St.Petersburg/8/2006) | PB1 | |
| CY035123 | A/NYMC X-163B (NYMC X-157-St.Petersburg/8/2006) | PA | |
| CY035118 | A/NYMC X-163B (NYMC X-157-St.Petersburg/8/2006) | HA | |
| CY035121 | A/NYMC X-163B (NYMC X-157-St.Petersburg/8/2006) | NP | |
| CY035120 | A/NYMC X-163B (NYMC X-157-St.Petersburg/8/2006) | NA | |
| CY035119 | A/NYMC X-163B (NYMC X-157-St.Petersburg/8/2006) | M | |
| CY035122 | A/NYMC X-163B (NYMC X-157-St.Petersburg/8/2006) | NS | |
| **H1N1-SOIV Wild Type and HYRs** | | | |
| CY044371 | A/New Jersey/11/1976 | PB1 | |
| CY044365 | A/New Jersey/11/1976 | HA | |
| CY044367 | A/New Jersey/11/1976 | NA | |
| CY062634 | A/X-53(Puerto Rico/8/1934-New Jersey/11/1976) | PB2 | |
| CY062633 | A/X-53(Puerto Rico/8/1934-New Jersey/11/1976) | PB1 | |
| CY062632 | A/X-53(Puerto Rico/8/1934-New Jersey/11/1976) | PA | |
| CY062627 | A/X-53(Puerto Rico/8/1934-New Jersey/11/1976) | HA | |
| CY062630 | A/X-53(Puerto Rico/8/1934-New Jersey/11/1976) | NP | |
| CY062629 | A/X-53(Puerto Rico/8/1934-New Jersey/11/1976) | NA | |
| CY062628 | A/X-53(Puerto Rico/8/1934-New Jersey/11/1976) | M | |
| CY062631 | A/X-53(Puerto Rico/8/1934-New Jersey/11/1976) | NS | |
| CY035037 | A/X-53a(Puerto Rico/8/1934-New Jersey/11/1976) | PB2 | |
| CY035036 | A/X-53a(Puerto Rico/8/1934-New Jersey/11/1976) | PB1 | |
| CY035035 | A/X-53a(Puerto Rico/8/1934-New Jersey/11/1976) | PA | |
| CY035030 | A/X-53a(Puerto Rico/8/1934-New Jersey/11/1976) | HA | |
| CY035033 | A/X-53a(Puerto Rico/8/1934-New Jersey/11/1976) | NP | |
| CY035032 | A/X-53a(Puerto Rico/8/1934-New Jersey/11/1976) | NA | |
| CY035031 | A/X-53a(Puerto Rico/8/1934-New Jersey/11/1976) | M | |
| CY035034 | A/X-53a(Puerto Rico/8/1934-New Jersey/11/1976) | NS | |
| **2009 H1N1pdm HYRs** | | | |
| CY058508 | A/NYMC X-179 (NYMC X-157-California/07/2009) | PB2 | |
| CY058509 | A/NYMC X-179 (NYMC X-157-California/07/2009) | PB1 | |
| CY058510 | A/NYMC X-179 (NYMC X-157-California/07/2009) | PA | |
| CY058511 | A/NYMC X-179 (NYMC X-157-California/07/2009) | HA | |
| CY058512 | A/NYMC X-179 (NYMC X-157-California/07/2009) | NP | |
| CY058513 | A/NYMC X-179 (NYMC X-157-California/07/2009) | NA | |
| CY058514 | A/NYMC X-179 (NYMC X-157-California/07/2009) | M | |
| CY058515 | A/NYMC X-179 (NYMC X-157-California/07/2009) | NS | |
| CY058516 | A/NYMC X-179A (NYMC X-157-California/07/2009) | PB2 | |
| CY058517 | A/NYMC X-179A (NYMC X-157-California/07/2009) | PB1 | |
| CY058518 | A/NYMC X-179A (NYMC X-157-California/07/2009) | PA | |
| CY058519 | A/NYMC X-179A (NYMC X-157-California/07/2009) | HA | |
| CY058520 | A/NYMC X-179A (NYMC X-157-California/07/2009) | NP | |
| CY058521 | A/NYMC X-179A (NYMC X-157-California/07/2009) | NA | |
| CY058522 | A/NYMC X-179A (NYMC X-157-California/07/2009) | M | |
| CY058523 | A/NYMC X-179A (NYMC X-157-California/07/2009) | NS | |
| **H2N2 Wild Type and HYRs** | | | |
| CY036822 | A/Korea/426/1968 | PB2 | |
| CY036821 | A/Korea/426/1968 | PB1 | |
| CY036815 | A/Korea/426/1968 | HA | |
| CY036818 | A/Korea/426/1968 | NP | |
| CY036817 | A/Korea/426/1968 | NA | |
| CY037302 | A/(Puerto Rico/8/1934-Korea/426/1968) | PB2 | |
| CY037301 | A/(Puerto Rico/8/1934-Korea/426/1968) | PB1 | |
| CY037300 | A/(Puerto Rico/8/1934-Korea/426/1968) | PA | |
| CY037295 | A/(Puerto Rico/8/1934-Korea/426/1968) | HA | |
| CY037298 | A/(Puerto Rico/8/1934-Korea/426/1968) | NP | |
| CY037297 | A/(Puerto Rico/8/1934-Korea/426/1968) | NA | |
| CY037296 | A/(Puerto Rico/8/1934-Korea/426/1968) | M | |
| CY037299 | A/(Puerto Rico/8/1934-Korea/426/1968) | NS | |
| CY044331 | A/Japan/305/1957 | PB1 | |
| CY044325 | A/Japan/305/1957 | HA | |
| CY044328 | A/Japan/305/1957 | NP | |
| CY044327 | A/Japan/305/1957 | NA | |
| CY044324 | A/X-135(Puerto Rico/8/1934-Japan/305/1957) | PB2 | |
| CY044323 | A/X-135(Puerto Rico/8/1934-Japan/305/1957) | PB1 | |
| CY044322 | A/X-135(Puerto Rico/8/1934-Japan/305/1957) | PA | |
| CY044317 | A/X-135(Puerto Rico/8/1934-Japan/305/1957) | HA | |
| CY044320 | A/X-135(Puerto Rico/8/1934-Japan/305/1957) | NP | |
| CY044319 | A/X-135(Puerto Rico/8/1934-Japan/305/1957) | NA | |
| CY044318 | A/X-135(Puerto Rico/8/1934-Japan/305/1957) | M | |
| CY044321 | A/X-135(Puerto Rico/8/1934-Japan/305/1957) | NS | |
| **H3N2 Wild Type and HYRs** | | | |
| CY044308 | A/X-31B(Puerto Rico/8.1934-Aichi/2/1968) | | PB2 |
| CY044307 | A/X-31B(Puerto Rico/8.1934-Aichi/2/1968) | | PB1 |
| CY044306 | A/X-31B(Puerto Rico/8.1934-Aichi/2/1968) | | PA |
| CY044301 | A/X-31B(Puerto Rico/8.1934-Aichi/2/1968) | | HA |
| CY044304 | A/X-31B(Puerto Rico/8.1934-Aichi/2/1968) | | NP |
| CY044303 | A/X-31B(Puerto Rico/8.1934-Aichi/2/1968) | | NA |
| CY044305 | A/X-31B(Puerto Rico/8.1934-Aichi/2/1968) | | M |
| CY044302 | A/X-31B(Puerto Rico/8.1934-Aichi/2/1968) | | NS |
| CY033606 | A/Beijing/32/1992 | | HA |
| CY033608 | A/Beijing/32/1992 | | NA |
| CY036814 | A/X-117(Puerto Rico/8/1934-Beijing/32/1992) | | PB2 |
| CY036813 | A/X-117(Puerto Rico/8/1934-Beijing/32/1992) | | PB1 |
| CY036812 | A/X-117(Puerto Rico/8/1934-Beijing/32/1992) | | PA |
| CY036807 | A/X-117(Puerto Rico/8/1934-Beijing/32/1992) | | HA |
| CY036810 | A/X-117(Puerto Rico/8/1934-Beijing/32/1992) | | NP |
| CY036809 | A/X-117(Puerto Rico/8/1934-Beijing/32/1992) | | NA |
| CY036808 | A/X-117(Puerto Rico/8/1934-Beijing/32/1992) | | M |
| CY036811 | A/X-117(Puerto Rico/8/1934-Beijing/32/1992) | | NS |
| CY034115 | A/Wyoming/03/2003 | | PB2 |
| CY034114 | A/Wyoming/03/2003 | | PB1 |
| CY034113 | A/Wyoming/03/2003 | | PA |
| CY034108 | A/Wyoming/03/2003 | | HA |
| CY034110 | A/Wyoming/03/2003 | | NA |
| CY033637 | A/NYMC X-149C6(Puerto Rico/8/1934-Wyoming/03/2003) | | PB2 |
| CY033636 | A/NYMC X-149C6(Puerto Rico/8/1934-Wyoming/03/2003) | | PB1 |
| CY033635 | A/NYMC X-149C6(Puerto Rico/8/1934-Wyoming/03/2003) | | PA |
| CY033630 | A/NYMC X-149C6(Puerto Rico/8/1934-Wyoming/03/2003) | | HA |
| CY033633 | A/NYMC X-149C6(Puerto Rico/8/1934-Wyoming/03/2003) | | NP |
| CY033632 | A/NYMC X-149C6(Puerto Rico/8/1934-Wyoming/03/2003) | | NA |
| CY033631 | A/NYMC X-149C6(Puerto Rico/8/1934-Wyoming/03/2003) | | M |
| CY033634 | A/NYMC X-149C6(Puerto Rico/8/1934-Wyoming/03/2003) | | NS |
| CY033644 | A/New York/55/2004 | | PB1 |
| CY033638 | A/New York/55/2004 | | HA |
| CY033640 | A/New York/55/2004 | | NA |
| CY095717 | A/NYMC X-157 (Puerto Rico/8/1934-New York/55/2004) | | PB2 |
| CY095716 | A/NYMC X-157 (Puerto Rico/8/1934-New York/55/2004) | | PB1b |
| CY095715 | A/NYMC X-157 (Puerto Rico/8/1934-New York/55/2004) | | PB1a |
| CY095714 | A/NYMC X-157 (Puerto Rico/8/1934-New York/55/2004) | | PA |
| CY095709 | A/NYMC X-157 (Puerto Rico/8/1934-New York/55/2004) | | HA |
| CY095712 | A/NYMC X-157 (Puerto Rico/8/1934-New York/55/2004) | | NP |
| CY095711 | A/NYMC X-157 (Puerto Rico/8/1934-New York/55/2004) | | NA |
| CY095710 | A/NYMC X-157 (Puerto Rico/8/1934-New York/55/2004) | | M |
| CY095713 | A/NYMC X-157 (Puerto Rico/8/1934-New York/55/2004) | | NS |
| CY035013 | A/NYMC X-157A (Puerto Rico/8/1934-New York/55/2004) | | PB2 |
| CY035012 | A/NYMC X-157A (Puerto Rico/8/1934-New York/55/2004) | | PB1 |
| CY035011 | A/NYMC X-157A (Puerto Rico/8/1934-New York/55/2004) | | PA |
| CY035006 | A/NYMC X-157A (Puerto Rico/8/1934-New York/55/2004) | | HA |
| CY035009 | A/NYMC X-157A (Puerto Rico/8/1934-New York/55/2004) | | NP |
| CY035008 | A/NYMC X-157A (Puerto Rico/8/1934-New York/55/2004) | | NA |
| CY035007 | A/NYMC X-157A (Puerto Rico/8/1934-New York/55/2004) | | M |
| CY035010 | A/NYMC X-157A (Puerto Rico/8/1934-New York/55/2004) | | NS |
| CY035117 | A/NYMC X-157B (Puerto Rico/8/1934-New York/55/2004) | | PB2 |
| CY035116 | A/NYMC X-157B (Puerto Rico/8/1934-New York/55/2004) | | PB1 |
| CY035115 | A/NYMC X-157B (Puerto Rico/8/1934-New York/55/2004) | | PA |
| CY035110 | A/NYMC X-157B (Puerto Rico/8/1934-New York/55/2004) | | HA |
| CY035113 | A/NYMC X-157B (Puerto Rico/8/1934-New York/55/2004) | | NP |
| CY035112 | A/NYMC X-157B (Puerto Rico/8/1934-New York/55/2004) | | NA |
| CY035111 | A/NYMC X-157B (Puerto Rico/8/1934-New York/55/2004) | | M |
| CY035114 | A/NYMC X-157B (Puerto Rico/8/1934-New York/55/2004) | | NS |
| CY034123 | A/Wisconsin/67/2005 | | PB2 |
| CY034122 | A/Wisconsin/67/2005 | | PB1 |
| CY034121 | A/Wisconsin/67/2005 | | PA |
| CY034116 | A/Wisconsin/67/2005 | | HA |
| CY034119 | A/Wisconsin/67/2005 | | NP |
| CY034118 | A/Wisconsin/67/2005 | | NA |
| CY034120 | A/Wisconsin/67/2005 | | NS |
| CY033653 | A/NYMC X-161 (Puerto Rico/8/1934-Wisconsin/67/2005) | | PB2 |
| CY033652 | A/NYMC X-161 (Puerto Rico/8/1934-Wisconsin/67/2005) | | PB1 |
| CY033651 | A/NYMC X-161 (Puerto Rico/8/1934-Wisconsin/67/2005) | | PA |
| CY033646 | A/NYMC X-161 (Puerto Rico/8/1934-Wisconsin/67/2005) | | HA |
| CY033649 | A/NYMC X-161 (Puerto Rico/8/1934-Wisconsin/67/2005) | | NP |
| CY033648 | A/NYMC X-161 (Puerto Rico/8/1934-Wisconsin/67/2005) | | NA |
| CY033647 | A/NYMC X-161 (Puerto Rico/8/1934-Wisconsin/67/2005) | | M |
| CY033650 | A/NYMC X-161 (Puerto Rico/8/1934-Wisconsin/67/2005) | | NS |
| CY036918 | A/NYMC X-161A (Puerto Rico/8/1934-Wisconsin/67/2005) | | PB2 |
| CY036917 | A/NYMC X-161A (Puerto Rico/8/1934-Wisconsin/67/2005) | | PB1 |
| CY036916 | A/NYMC X-161A (Puerto Rico/8/1934-Wisconsin/67/2005) | | PA |
| CY036911 | A/NYMC X-161A (Puerto Rico/8/1934-Wisconsin/67/2005) | | HA |
| CY036914 | A/NYMC X-161A (Puerto Rico/8/1934-Wisconsin/67/2005) | | NP |
| CY036913 | A/NYMC X-161A (Puerto Rico/8/1934-Wisconsin/67/2005) | | NA |
| CY036912 | A/NYMC X-161A (Puerto Rico/8/1934-Wisconsin/67/2005) | | M |
| CY036915 | A/NYMC X-161A (Puerto Rico/8/1934-Wisconsin/67/2005) | | NS |
| CY045843 | A/NYMC X-161B (Puerto Rico/8/1934-Wisconsin/67/2005) | | PB2 |
| CY045842 | A/NYMC X-161B (Puerto Rico/8/1934-Wisconsin/67/2005 | | PB1 |
| CY045841 | A/NYMC X-161B (Puerto Rico/8/1934-Wisconsin/67/2005 | | PA |
| CY045836 | A/NYMC X-161B (Puerto Rico/8/1934-Wisconsin/67/2005 | | HA |
| CY045839 | A/NYMC X-161B (Puerto Rico/8/1934-Wisconsin/67/2005 | | NP |
| CY045838 | A/NYMC X-161B (Puerto Rico/8/1934-Wisconsin/67/2005 | | NA |
| CY045837 | A/NYMC X-161B (Puerto Rico/8/1934-Wisconsin/67/2005 | | M |
| CY045840 | A/NYMC X-161B (Puerto Rico/8/1934-Wisconsin/67/2005 | | NS |
| CY035029 | A/Brisbane/10/2007 | | PB2 |
| CY035028 | A/Brisbane/10/2007 | | PB1 |
| CY035027 | A/Brisbane/10/2007 | | PA |
| CY035022 | A/Brisbane/10/2007 | | HA |
| CY035025 | A/Brisbane/10/2007 | | NP |
| CY035024 | A/Brisbane/10/2007 | | NA |
| CY035026 | A/Brisbane/10/2007 | | NS |
| CY035021 | A/NYMC X-171(Puerto Rico/9/1934-Brisbane/10/2007) | | PB2 |
| CY035020 | A/NYMC X-171(Puerto Rico/9/1934-Brisbane/10/2007) | | PB1 |
| CY035019 | A/NYMC X-171(Puerto Rico/9/1934-Brisbane/10/2007) | | PA |
| CY035014 | A/NYMC X-171(Puerto Rico/9/1934-Brisbane/10/2007) | | HA |
| CY035017 | A/NYMC X-171(Puerto Rico/9/1934-Brisbane/10/2007) | | NP |
| CY035016 | A/NYMC X-171(Puerto Rico/9/1934-Brisbane/10/2007) | | NA |
| CY035015 | A/NYMC X-171(Puerto Rico/9/1934-Brisbane/10/2007) | | M |
| CY035018 | A/NYMC X-171(Puerto Rico/9/1934-Brisbane/10/2007) | | NS |
| CY035141 | A/NYMC X-171A(Puerto Rico/9/1934-Brisbane/10/2007) | | PB2 |
| CY035140 | A/NYMC X-171A(Puerto Rico/9/1934-Brisbane/10/2007) | | PB1 |
| CY035139 | A/NYMC X-171A(Puerto Rico/9/1934-Brisbane/10/2007) | | PA |
| CY035134 | A/NYMC X-171A(Puerto Rico/9/1934-Brisbane/10/2007) | | HA |
| CY035137 | A/NYMC X-171A(Puerto Rico/9/1934-Brisbane/10/2007) | | NP |
| CY035136 | A/NYMC X-171A(Puerto Rico/9/1934-Brisbane/10/2007) | | NA |
| CY035135 | A/NYMC X-171A(Puerto Rico/9/1934-Brisbane/10/2007) | | M |
| CY035138 | A/NYMC X-171A(Puerto Rico/9/1934-Brisbane/10/2007) | | NS |
| CY035149 | A/NYMC X-171B(Puerto Rico/9/1934-Brisbane/10/2007) | | PB2 |
| CY035148 | A/NYMC X-171B(Puerto Rico/9/1934-Brisbane/10/2007) | | PB1 |
| CY035147 | A/NYMC X-171B(Puerto Rico/9/1934-Brisbane/10/2007) | | PA |
| CY035142 | A/NYMC X-171B(Puerto Rico/9/1934-Brisbane/10/2007) | | HA |
| CY035145 | A/NYMC X-171B(Puerto Rico/9/1934-Brisbane/10/2007) | | NP |
| CY035144 | A/NYMC X-171B(Puerto Rico/9/1934-Brisbane/10/2007) | | NA |
| CY035143 | A/NYMC X-171B(Puerto Rico/9/1934-Brisbane/10/2007) | | M |
| CY035146 | A/NYMC X-171B(Puerto Rico/9/1934-Brisbane/10/2007) | | NS |
| CY058500 | A/NYMC X-175C(Puerto Rico/9/1934-Brisbane/10/2007) | | PB2 |
| CY058501 | A/NYMC X-175C(Puerto Rico/9/1934-Brisbane/10/2007) | | PB1 |
| CY058502 | A/NYMC X-175C(Puerto Rico/9/1934-Brisbane/10/2007) | | PA |
| CY058503 | A/NYMC X-175C(Puerto Rico/9/1934-Brisbane/10/2007) | | HA |
| CY058504 | A/NYMC X-175C(Puerto Rico/9/1934-Brisbane/10/2007) | | NP |
| CY058505 | A/NYMC X-175C(Puerto Rico/9/1934-Brisbane/10/2007) | | NA |
| CY058506 | A/NYMC X-175C(Puerto Rico/9/1934-Brisbane/10/2007) | | M |
| CY058507 | A/NYMC X-175C(Puerto Rico/9/1934-Brisbane/10/2007) | | NS |
